# Prevalence and correlates of HIV infection among adolescents and young people living in fishing populations along Lake Victoria Fishing Communities in Uganda

**DOI:** 10.11604/pamj.2020.37.208.26124

**Published:** 2020-11-02

**Authors:** Gertrude Nanyonjo, Gershim Asiki, Ali Ssetaala, Teddy Nakaweesa, Mathias Wambuzi, Annet Nanvubya, Juliet Mpendo, Brenda Okech, Paul Kato Kitandwe, Leslie Nielsen, Annet Nalutaaya, Sabrina Welsh, Bernard Ssentalo Bagaya, Kundai Chinyenze, Pat Fast, Matt Price, Noah Kiwanuka

**Affiliations:** 1 UVRI/IAVI HIV Vaccine Program Limited, Entebbe, Uganda,; 2 African Population and Health Research Center, Nairobi, Kenya,; 3 MRC/UVRI and LSHTM Uganda Research, Entebbe, Uganda,; 4 Partnership for Research on Ebola Virus in Liberia (PREVAIL), Monrovia, Liberia,; 5 Makerere University, College of Health Sciences, Uganda Tuberculosis and Implementation Research Consortium, Kampala, Uganda,; 6 Human Vaccines Project, New York, NY, USA,; 7 Department of Immunology and Molecular Biology, School of Biomedical Sciences, College of Health Sciences, Makerere University, Kampala, Uganda,; 8 IAVI, New York, NY, USA,; 9 Department of Epidemiology and Biostatistics, University of California at San Francisco, San Francisco, CA, USA,; 10 Department of Epidemiology and Biostatistics, School of Public Health, College of Health Sciences, Makerere University, Kampala, Uganda

**Keywords:** Adolescents, young people, HIV, prevalence, fishing, community, Uganda

## Abstract

**Introduction:**

fishing communities in Uganda are key populations for HIV, with persistently higher prevalence and incidence than the general population.

**Methods:**

between March and August 2014, a cross sectional survey was conducted in 10 fishing communities of Lake Victoria in Uganda. Data was collected on socio-behavioural characteristics using interviewer administered questionnaires and venous blood collected for HIV testing. Prevalent HIV infections among adolescents and young people aged 13 to 24 years was estimated and the factors associated with those infections determined using multi variable logistic regression modelling.

**Results:**

HIV prevalence was 10.8% among the 630 (96.5%) who provided a blood sample. Females were 3.5 times as likely to have HIV infection as males (aOR=3.52, 95% CI: 1.34-9.22). Young people aged 20-24 years were twice as likely to be HIV infected as those aged 13-19 years (aOR=1.77, 95% CI: 0.05-2.10), participants without formal education or those who had studied up to primary level were more likely to be HIV infected than those who had post primary education ((aOR=2.45, 95% CI: 1.19-5.07) or (5.29 (1.35-20.71) respectively). Reporting more than one sexual partner in the past 6 months was associated with HIV prevalent infection than those reporting no sexual partners (aOR=6.44, 95% CI: 1.27-32.83).

**Conclusion:**

adolescents and young people aged 13-24 years in fishing communities around Lake Victoria, Uganda, have a high HIV prevalence, with females having a three-fold higher level than males. These findings highlight-the need to improve HIV prevention among young females living in these fishing communities.

## Introduction

The Human Immunodeficiency Virus (HIV), continues to be a major global public health and development challenge. HIV has claimed more than 35 million lives since its discovery in early 1980's. There were approximately 37.9 million people living with HIV at the end of 2018 with 1.7 million people becoming newly infected in 2018 globally [1]. It is estimated that more than half of new HIV infections worldwide occur among young people of 15-24 years and up to a third of people living with HIV/AIDS are 25 years or younger [2]. Young people are more disproportionally affected by the HIV/AIDS epidemic in sub-Saharan Africa. The Uganda population-based HIV impact assessment indicated that HIV prevalence is nearly three times higher in young Ugandans aged 20-24 compared to those aged 15-19 [3]. HIV prevalence is almost three times higher among females than males aged 15 to 24 [4].

Fishing communities (FCs) have higher HIV prevalence and incidence rates than the general population in Uganda, and are identified as a most at risk population [5-10]. Commercial fishing attracts a significant proportion of young people for gainful employment to the shores and islands of Lake Victoria. A study in fishing communities along Lake Victoria, Uganda revealed that that young people aged 13-24 years had very high HIV incidence (7.5 /100 person years at risk) [11]. HIV prevalence among young women aged 15-24 years and men aged 20-29 years in fishing communities was also reported to be higher than that of young people in the general communities in Rakai [12,13]. However, in these studies, young people were under-represented, and the studies were not adequately powered to investigate risk factors for HIV among young people. Most HIV studies conducted in fishing communities targeted participants from the age of 18 years or did not include sufficient numbers of young people to explore HIV prevalence and related factors among a younger age group.

Young people are a special group transitioning from childhood to adulthood and this evolution may be associated with increased curiosity and exploration of new sexual and other behaviours like alcohol consumption. Fishing communities are often characterised by high mobility, a large presence of bars, lodges and entertainment halls, commercial sex work, and limited access to education and health care services [13,14]. The nature of fishing entails regular cash transactions that may make it easier for young residents to have access to bars and/or commercial sex. Fishing communities also have a high number of emancipated minors. These factors could potentially elevate the risk of HIV infection among young residents in FCs. HIV prevalence and associated characteristics among young people aged 13-24 years living in 10FCs along the shores of Lake Victoria, Uganda was explored to inform prevention efforts in this age group.

## Methods

**Study design:** this was a cross sectional baseline survey of a larger observational cohort titled the Community HIV Epidemiological and Social Behavioural Survey (CHESS). This study was conducted to characterize persons living in Ugandan FCs and assess the determinants of HIV vulnerability. CHESS included community mapping and census, and households were selected at random for recruitment. Eligible participants were aged 13-49 years, having stayed in the community for at least 6 months and included anyone at home in the selected household. Selection was not based on any HIV risk criteria. Apart from laboratory assays, all study procedures were conducted in the community research hubs.

**Study setting:** the study was conducted in 10 fishing communities of the Lake Victoria Basin. The study included two mainland communities along the lake shore and eight island ones in Kalangala, Wakiso and Mukono districts in Uganda. Selection was based on population size of between 500 and 1000 people. A fishing community is a group of persons living in a village or trading centre that is adjacent to a lake landing site where the predominant economic activity is fishing [7]. Inhabitants of these communities are diverse and usually include fishermen, boat crew, boat owners, boat makers, boat repairers, fish processors, fish traders, shop keepers, shop owners and workers of bars/restaurants/lodges as well as sex workers [15]. They live in informal clustered settlements that are often densely populated and many tend to be highly mobile, often following the fish as they migrate seasonally [16].

**Study population:** this analysis was limited to younger CHESS participants, aged 13-24 years.

### Data collection and analysis

**Data collection:** in each study community, mapping and a household enumeration census were conducted to generate a sampling frame that was used for random selection of households. All eligible participants in selected households who provided consent and assent in case of un-emancipated minors were interviewed. HIV testing was performed after counselling by qualified medical personnel. Data on demographics, behavioural characteristics, HIV risk behaviour and HIV testing were collected through face-to-face interviews using a structured questionnaire. HIV risk behaviour included alcohol consumption, new sexual partners in previous six months, unprotected sexual intercourse with more than one partner or a new sexual partner in the previous six months. Data collection forms were checked for completeness, accuracy and consistency by field-based reviewers who ensured that errors were corrected in the field. Double data entry was carried out using Microsoft Access (version 11.0, Redmond, WA, USA) entry screens with range, consistency, and logic checks. Corrections were made in case of any discrepancies between the two entries by referring to the source documents. Data was backed-up on both the server and external hard drives.

**Laboratory testing:** rapid HIV tests were performed in the community by certified laboratory technologists as per the Uganda National HIV Testing Algorithm (18). All blood samples were first tested on Alere Determine HIV 1/2 (Alere Medical Co, Ltd., Chiba, Japan), and if negative, results were reported as negative. All determine-positive samples were further tested using HIV 1/2 Stat-Pak assay (Chembio Diagnostic Systems, Inc. NY, USA), and if also positive, results were reported as positive. However, if negative on Stat-Pak, Uni-Gold HIV (Trinity Biotech, Bray, Ireland) was used as a tiebreaker. As an additional quality control measure, all positive rapid results were retested using two parallel enzyme-linked immunosorbent assay (EIA) tests: Vironostika HIV Ag/Ab, (BioMérieux SA.Marcy L'Etoile, France) and Murex HIV1.2.0 (Murex Biotech Limited, Dartford, UK). Discordant EIA results were resolved using real time polymerase chain reaction (COBAS TaqMan HIV-1, Roche Molecular Diagnostics, and Pleasanton CA, USA).

**Data analysis:** the dependent variable was prevalent HIV infection. Independent variables selected a priori based on previous research in these communities were age, sex, occupation, education level and length of stay in the community. Characteristics of the study population were described using mean-standard deviation, median and interquartile range for continuous and discrete variables, and proportions for categorical ones. For the multivariable analysis, variables predicting prevalent HIV infection were selected based on previous literature or biological plausibility and factors significant at p-value <0.1 on bivariate analysis. Multivariable logistic regression was used to estimate adjusted odds ratios to determine factors associated with prevalent HIV infection. The likelihood ratio test was used to determine fitness of the predictors in the model. Data was analysed using STATA (version 13, Stata Corp, College Station, Texas, USA).

**Ethical considerations:** ethical approval was obtained from the Uganda Virus Research Institute Research Ethics Committee (UVRI-REC, FWA number 00001354) and the Uganda National Council for Science and Technology (UNCST, FWA number 00001293). Participants were given detailed information about the study through an information sheet read to them in the language they understood best that was either English or Luganda. Documented informed consent was thought for participants aged 18-24 years and emancipated minors. Participants aged less that 18 years were enrolled following documented assent and consent from guardians.

## Results

**Characteristics of the study participants:** a total of 656 young people aged 13-24 years were interviewed from March 2014 to August 2014. The median (IQR) age was 21 (18-23) years and 57.3% (376/656) were adolescent girls and young women. About half (52.7%, 346/656) had attained primary level education, and over one third had attained beyond primary level of education; 105 (16.0%) reported being students at the time of the study. Under half (47.8%) reported being married; nearly 8% reported being in a polygamous marriage. Over a quarter (27.4%) were engaged in fishing related activities including fishing, fish mongering, fish processing and/or fish smoking, and boat making. Approximately 25% reported no sexual partners in the past 6 months, while 20% reported more than one sexual partner in that time. Among the sexually active participants, about 9% reported consistent condom use. Age at first sex, or if participant had never had sex was not assessed. Majority (85. 3%) had stayed in the community for more than 1 year and among the housewives, 70.8% were living on the Island (Table 1, Table 2).

**Table 1:** socio-demographic characteristics of young people by residence

Demographic characteristics	Total (n=656)		Lake shore (n=222)		Island (n=434)		p-value
	n	(%)	n	(%)	n	(%)	
**Age (years)**							
Median (IQR)	21(18 - 23)		19.5 (17 - 22)		21 (19 - 23)		
13-19	236	(36.0)	111	47.0	125	53.0	<0.001
20-24	420	(64.0)	111	26.4	309	73.6	
**Gender**							
Male	280	(42.7)	87	31.1	193	68.9	0.196
Female	376	(57.3)	135	35.9	241	64.1	
Tribe^*^							
Muganda	315	(48.0)	116	36.8	199	63.2	0.120
Munyankole/Munyarwanda/Murundi/Mukiga	119	(18.1)	36	30.3	83	69.7	
Musoga	67	(10.2)	28	41.8	39	58.2	
Others	154	(23.5)	42	27.3	112	72.7	
Missing	1	(0.2)	0	0.0	1	100.0	
**Religion§**							
Roman Catholic/Protestant	409	(62.3)	140	34.2	269	65.8	0.617
Muslim	150	(22.9)	46	30.7	104	69.3	
Other#	96	(14.6)	36	37.5	60	62.5	
Missing	1	(0.2)	0	0.0	1	100.0	
**Highest education level**							
No formal	21	(3.2)	3	14.3	18	85.7	0.009
Primary	346	(52.7)	105	30.3	241	69.7	
Post Primary	289	(44.1)	114	39.4	175	60.6	
**Occupation**							
Fishing/Fish-related	180	(27.4)	22	12.2	158	87.8	<0.001
Trade/Business	64	(9.8)	25	39.1	39	60.9	
Bar/Lodge/Restaurant attendant	57	(8.7)	16	28.1	41	71.9	
Student	105	(16.0)	67	63.8	38	36.2	
Housewife	96	(14.6)	28	29.2	68	70.8	
Other^!^	154	(23.5)	64	41.6	90	58.4	
**Marital status**							
Not currently married	342	(52.1)	153	44.7	189	55.3	<0.001
Married monogamous	262	(39.9)	57	21.8	205	78.2	
Married polygamous	52	(7.9)	12	23.1	40	76.9	

* 1 missing tribe, Tanzanian, Kenyan, Mugishu, Langi, Musamya, Alur, Itesot, Munyoli, Mudama, Karamajong, Japhadhola, Mugwere, Kebu, Mudama, Congolese, Mufumbira, § 1 missing religion, ! Bar/Lodge/Restaurant owner, Agriculture/Farming, Government employee, Medical worker(non-government), Loading and off-loading, making ropes, Unemployed, Boda boda cyclist

**Table 2:** behavioural characteristics and HIV status of young people by residence

Characteristics	Total (n=656)		Lake shore (n=222)		Island (n=434)		p-value
	n	(%)	n	(%)	n	(%)	
**Sexual partners in past 6 months**							
None	162	(24.7)	90	55.6	72	44.4	<0.001
One partner	360	(54.9)	107	29.7	253	70.3	
More than one partner	134	(20.4)	25	18.7	109	81.3	
New partners in past 6 months							
None	334	(67.6)	95	28.4	239	71.6	0.046
One partner	115	(23.3)	32	27.8	83	72.2	
More than one partner	45	(9.1)	5	11.1	40	88.9	
**Condom use in past 6 months**							
Never/Sometimes/Inconsistent	450	(91.1)	122	27.1	328	72.9	0.531
Always	44	(8.9)	10	22.7	34	77.3	
**Alcohol consumption in past 6 months**							
None	408	(62.2)	152	37.3	256	62.7	
Occasional/Regular	248	(37.8)	70	28.2	178	71.8	0.018
**Duration of stay in community^α^**							
Less than 1 year	84	(14.8)	24	28.6	60	71.4	<0.001
1 to 4 years	290	(51.1)	90	31.0	200	69.0	
More than 5 years	194	(34.2)	108	55.7	86	44.3	
HIV status^*^							
Negative	562	(85.7)	199	35.4	363	64.6	
Positive	68	(10.4)	12	17.6	56	82.4	
Missing	26	(4.0)	11	42.3	15	57.7	0.009

* 26 missing HIV status; a 88 missing duration of stay in community

### HIV prevalence and associated factors

**HIV prevalence among adolescents and young people:** among the 96% that were tested, HIV prevalence was 10.8% (68/630). Bivariate analysis showed that prevalence was markedly higher among females than males (15.4% vs 4.8%, p<0.001), those aged 20-24 years compared to those aged 13-19(14.3% vs 4.8%, p<0.001),house wives (24.2% vs 23.2%, p<0.001), participants in monogamous marriages compared to those in polygamous marriages (16.5% vs13.5%, p<0.001), among regular alcohol consumers compared to non-alcohol consumers(16.7% vs 7.3%, p<0.001) and those who had more than one sexual partner in the past 6 months compared to those with one sexual partner (17.2% vs 12.6%, p<0.001).

**Factors correlated with prevalent HIV infection among adolescents and young people:** when controlling for other factors in multivariable analysis, female participants were 3.5 times more likely to have prevalent HIV infection compare to male participants (aOR=3.5,95% CI: 1.34-9.22). Participants older than 19 years, lacking formal education or primary level education compared to those with greater than primary level education, having more than one sexual partner in the past six months compared to those reporting no sexual partners were associated with prevalent HIV infection (Table 3). It was observed that duration of stay in the community was not significantly associated with prevalent HIV infection; duration of stay and to a lesser degree, volunteer occupation appeared to confound the association of both education and sexual partners with the outcome and retaining duration and occupation in the analysis provided a better predictive model of HIV infection. The HIV prevalence among those who always reported condom use was low (2.4%, 1/41) and due to these small numbers, this variable was not assessed in the multivariable analysis.

**Table 3:** HIV prevalence, unadjusted (cOR) and adjusted odds ratios (aORs) of factors associated with prevalent HIV infection among study participants (n=630)

Characteristics	Total	HIV-positive		P-value	Unadjusted Odds Ratio		Adjusted Odds Ratio		
		(n=68)							
		n	%		cOR	(95% CI)	aOR	(95% CI)	P-value
**Age**									
13-19	230	11	4.8	<0.001	1 (Ref)		1 (Ref)		
20-24	400	57	14.3		3.31	(1.70 - 6.50)	2.72	(1.15 - 6.39)	0.022
**Gender**									
Male	272	13	4.8	<0.001	1 (Ref)		1 (Ref)		
Female	358	55	15.4		3.62	(1.93 - 6.77)	3.52	(1.34 - 9.22)	0.011
Tribe^*^									
Muganda	303	27	8.9	0.116	1 (Ref)				
Munyankole/Munyarwanda/ Murundi/Mukiga	113	18	15.9		1.94	(1.02 - 3.67)			
Musoga	62	4	26.5		0.7	(0.24 - 2.09)			
Others^¶^	151	19	12.6		1.47	(0.79 - 2.74)			
**Religion§**									
Roman Catholic/ Protestant(Anglican)	391	49	12.5	0.038	1 (Ref)				
Muslim	147	16	10.9		0.85	(0.47 - 1.55)			
Other^#^	91	3	3.3		0.24	(0.07 - 0.78)			
**Highest education level**									
Post Primary	274	19	6.9	0.005	1 (Ref)		1 (Ref)		
Primary	337	44	13.1		2.02	(0.15 - 3.54)	2.45	(1.19 - 5.07)	0.015
No formal education	19	5	26.3		4.79	(1.56 - 14.73)	5.29	(1.35 - 20.71)	0.017
**Occupation**									
Other!	479	32	6.7	<0.001	1 (Ref)		1 (Ref)		
Bar/Lodge/Restaurant attendant	56	13	23.2		4.22	(2.06 - 8.64)	1.38	(0.45 - 4.21)	0.57
Housewife	95	23	24.2		4.46	(2.47 - 8.06)	1.92	(0.89 - 4.16)	0.098
**Duration of stay in community^α^**									
Less than 1 year	81	11	13.6	0.065	1 (Ref)		1 (Ref)		
1 to 4 years	278	28	10.1		0.71	(0.34 - 1.50)	0.70	(0.31 - 1.60)	0.402
More than 5 years	186	10	5.4		0.36	(0.15 - 0.89)	0.56	(0.21 - 1.52)	0.256
**Marital status**									
Not currently married	323	19	5.9	<0.001	1 (Ref)				
Married monogamous	255	42	16.5		3.15	(1.79 - 5.58)			
Married polygamous	52	7	13.5		2.49	(0.99 - 6.25)			
**Sexual partners in past 6 months**									
None	153	2	1.3	<0.001	1 (Ref)		1 (Ref)		
One partner	349	44	12.6		10.9	(2.61 -45.53)	3.16	(0.68 - 14.73)	0.143
More than one partner	128	22	17.2		15.7	(3.61 - 68.06)	6.44	(1.27 - 32.83)	0.025
**New partners in past 6 months**									
None	321	48	15	0.39	1 (Ref)				
One partner	111	11	9.9		0.63	(0.31 - 1.25)			
More than one partner	45	7	15.6		1.05	(0.44 - 2.48)			
**Condom use in past 6 months**									
Never/Sometimes/Inconsistent	436	65	14.9	0.027	1 (Ref)				
Always	41	1	2.4		0.14	(0.02 - 1.06)			
**Alcohol consumption in past 6 months**									
None	396	29	7.3	<0.001	1 (Ref)				
Occasional/Regular	234	39	16.7		2.53	(1.52 - 4.22)			
**Residence**									
Mainland Lake shore	211	12	5.7	0.003	1 (Ref)				
Island	419	56	13.4		2.56	(1.34 - 4.89)			

* 1 missing tribe; Tanzanian, Kenyan; § 1 missing religion; # Born Again/Pentecostal, Seventh Day Advent, Traditional, No religion; ! Fishing related activities, trade and business, Student, Agriculture/Farming, Mechanic, Construction, Loading and off-loading; α 85 missing duration of stay in community

## Discussion

The study found a high prevalence of HIV infection (10.8%) among adolescent and young people in fishing communities of Lake Victoria, Uganda. In this population, females and those with less than eight years of formal education and those with more than one sexual partner were more likely to have prevalent HIV infection. HIV prevalence among this population is almost three times higher than that of young people in the general Ugandan population (3.7%) [17]. This finding is consistent with findings of other studies conducted in similar fishing communities in Uganda that have reported HIV prevalence ranging from 10-20% in participants as young as 15 years [4,6,7,18,19]. The HIV prevalence in FCs could be due to mobility of the population based on seasonal fishing. The odds of prevalent HIV were high among those aged 20-24 years compared to younger participants. Increasing prevalence with age is not surprising as sexual activity can increase and antiretroviral therapy (ART) access can improve longevity of those with HIV infection. In previous studies, only 12 to 13% of young people reported that they had sex before age 15; and by age 18, 60% of young women and 47 percent of young men had initiated sexual activity [20,21]. However, this study did not assess the age of sexual debut. The findings of this study are consistent with other studies in sub-Saharan Africa where adolescent girls are reported to be three to four times more likely than adolescent boys to be infected with HIV [22].

Studies in African fishing communities, specifically in Kenya, Uganda and Malawi have shown that young women in fishing communities are at higher risk of HIV than their male counterparts due to early sexual activity, engagement in transactional sex in the form of sex-for-fish or sex-for-money and other gifts [4,6,19,20,23,24]. This study among young people of 13-24 years of Lake Victoria fishing communities revealed similar findings, with HIV prevalence among young females being more than three times higher than that among male counterparts [6,25]. Gender inequalities including gender-based violence, socio-economic vulnerability, male child preference, and early marriages, exacerbate women´s physiological vulnerability to HIV and limit access to HIV services [19,26]. Additionally, high HIV prevalence remains in fishing communities due to Fisher-folks engaging in high-risk sex as evidenced in studies conducted in Uganda [4]. Of note among this study population, HIV prevalence decreased with increasing levels of education. However, this has been an inconsistent observation in the literature [4]. Attaining education may increase opportunities or willingness and/or ability to access health information as well as the comprehension of this health information; the time required for school activities and the potential discipline could delay sexual debut thus reducing the risk of HIV acquisition. This finding is consistent with previous findings in fishing population and the general population [6,18,20].

The prevalence of HIV remains high in fishing communities due to high risk behaviours as evidenced by our findings in this study. Risky sexual behaviour was found to be common among young people in the fishing communities. This was also reported by Smolak [27] where a high proportion of fisher folk engage in sex with more than one partner. This agrees with this and other published findings because it was found that having more than one sexual partner was significantly associated with HIV infection [18], and not unexpectedly report of multiple partners was associated with prevalent HIV infection in our study as well [28]. These findings are consistent with previous studies in fishing communities hence the need for effective HIV prevention interventions for young people in these communities [7]. Being a housewife and employed in a bar, lodge or restaurant are associated with low finances and had borderline association with HIV infection. Though sex work in this population was not explicitly measured, employees in businesses earn meagre wages may supplement with their income with transactional sex activities, thus putting themselves at risk for HIV [11,13]. Consistent condom use was low and should be pointed out and re-emphasized among sexually active young people in these populations. There is high consumption of alcohol in fishing communities which is one of the drivers of HIV infection [29]. Excessive alcohol consumption may interfere with consistent condom use among sexually active young people hence increase I HIV infections.

This study had some limitations. It is possible that highly mobile young people could have been excluded and this could potentially lead to under-estimating HIV prevalence since mobility was found to be associated with HIV in other studies in fishing communities [11]. Previous work observed that mobility was associated with drop out [30-32] and this study specifically excluded participants who had been in the community for less than 6 months yet those had previously been observed to be at greater risk of dropping out. These findings may not be generalizable to all fishing communities in Uganda, around Lake Victoria, or elsewhere, however, a major strength of this study is the large sample size across many communities and the collection of detailed demographic and behavioural data. This study was cross sectional, and thus measured prevalent HIV at a single visit. While predictors of prevalent HIV infection may be useful for guiding interventions, understanding the current state of the epidemic and giving a crude indication of risk, conclusions about observed predictors of prevalent HIV and risk should be interpreted cautiously. A prospective cohort study design is better suited to understand risk factors of HIV acquisition, and further study is merited to understand the patterns of risks in youth residing in fishing communities.

## Conclusion

HIV prevalence among young people in this Ugandan FCs is three-fold higher than in the young people in general population. Young people in fishing communities of Lake Victoria are among marginalized groups. HIV prevalence is more among females than males hence females are at a higher risk of acquiring HIV. More HIV preventive interventions should focus on young people and may require special approaches to best reach young people and increase their uptake of these measures. Due to uniqueness of the social, economic, and behavioural characteristics of young people in the fishing communities, there is need to develop targeted, tailored HIV interventions to reach these populations.

### What is known about this topic

- Fishing communities (FCs) have higher incidence rates than the general population in Uganda;
- FCs are one of the key populations in Uganda.

### What this study adds

- Correlates of HIV infection in this population include female sex, age (20-24 years), primary level education and having more than one sexual partner.
